# Sexual dysfunction in men with multiple sclerosis

**DOI:** 10.1186/s13643-021-01643-3

**Published:** 2021-03-27

**Authors:** Lisa B. Grech, Michelle Allan, Barbora de Courten

**Affiliations:** 1 Department of Medicine, School of Clinical Sciences, Monash University, Clayton, 3168 Victoria Australia; 2 Melbourne School of Psychological Sciences, University of Melbourne, Parkville, Victoria 3010 Australia; 3 Department of Neurology, Monash Medical Centre, Monash Health, Clayton, Victoria 3168 Australia

We read with interest the review by Dastoorpoor and colleagues [1], highlighting the prevalence of sexual dysfunction in men with multiple sclerosis (MS). We congratulate the authors on bringing together research on the important topic of the high prevalence of sexual dysfunction, which may be caused, at least in part, by the high prevalence of psychological symptoms in MS, but may also precipitate or compound existing psychological symptoms and impact quality of life.

We would like to highlight the importance of contrasting work related to the prevalence of sexual dysfunction in MS against broader community samples when considering this research, given that sexual dysfunction in men is prevalent and increases with age and common ageing-related comorbidities (e.g. diabetes and cardiovascular disease) [2]. For example, one review highlighted that sexual dysfunction occurs in up to 52% of men in the community and that there is a positive relationship with increasing age [3]. A Turkish-based Internet survey reported 43.3% of male respondents experienced sexual dysfunction, with 72% in the 55–60-year-old range [4]. As the authors point out, some research into sexual dysfunction in men with MS has not shown an incidence increase with age as shown in community samples. A discussion about the prevalence of sexual dysfunction more broadly to distinguish MS-specific sexual dysfunction compared to dysfunction related to other comorbidities or ageing-related processes is warranted.

While a systematic review and meta-analysis are limited to available data from the original studies, we believe there is a need to consider sexual dysfunction across the spectrum of severity from mild through severe. The current review aptly pointed out the differences in measurement tools used across studies, but some discussion about the measurement of severity and its impact on prevalence estimates is warranted. A Danish community study found that 11% of male respondents reported sexual dysfunction (frequent and perceived as a problem), while 68% of males reported less severe sexual difficulties [5]. Many of the studies included in the review by Dastoorpoor and colleagues [1] reported on the severity, enabling a qualitative synthesis and discussion. Primary, secondary and tertiary causal aspects of sexual dysfunction were also reported in many of the included studies, again providing an opportunity for a nuanced discussion about the causal impact attributable to MS disease processes, the symptoms of MS or comorbid factors. Given the effort that goes into data search and screening for a systematic review, we encourage the authors to maximise the scientific value of their work with a secondary extraction and data synthesis to report on some of these aspects.

We again wish to thank the authors for bringing this research together and summarising information about the prevalence of sexual dysfunction for men with MS to date. We hope to have encouraged a follow-up article detailing some of the above aspects and that future reviews on this topic will be considered from the perspective of sexual dysfunction in men more broadly, across the severity of dysfunction and potential causal aspects to contextualise knowledge in this area. A more detailed understanding of this important clinical issue would assist with appropriate clinical attention and management, as well as referral for support to maximise the quality of life for men with MS who experience sexual dysfunction.
